# Functionalized Ionic Liquids-Modified Metal–Organic Framework Material Boosted the Enzymatic Performance of Lipase

**DOI:** 10.3390/molecules29102381

**Published:** 2024-05-18

**Authors:** Liran Ji, Wei Zhang, Yifei Zhang, Binbin Nian, Yi Hu

**Affiliations:** State Key Laboratory of Materials-Oriented Chemical Engineering, School of Pharmaceutical Sciences, Nanjing Tech University, Nanjing 210009, China; 202261218214@njtech.edu.cn (L.J.); zhangwei218009@163.com (W.Z.); 202161109038@njtech.edu.cn (Y.Z.)

**Keywords:** metal–organic framework, ionic liquids modification, lipase immobilization

## Abstract

The development of immobilized enzymes with high activity and stability is critical. Metal–organic frameworks (MOFs) have attracted much academic and industrial interest in the field of enzyme immobilization due to their unique properties. In this study, the amino-functionalized ionic liquid (NIL)-modified metal–organic framework (UiO-66-NH_2_) was prepared to immobilize *Candida rugosa* lipase (CRL), using dialdehyde starch (DAS) as the cross-linker. The results of the Fourier transform infrared (FT-IR) spectra, X-ray powder diffraction (XRD), and scanning electronic microscopy (SEM) confirmed that the NIL was successfully grafted to UiO-66-NH_2_. The CRL immobilized on NIL-modified UiO-66-NH_2_ (UiO-66-NH_2_-NIL-DAS@CRL) exhibited satisfactory activity recovery (79.33%), stability, reusability, and excellent organic solvent tolerance. The research results indicated that ionic liquid-modified UiO-66-NH_2_ had practical potential for application in enzyme immobilization.

## 1. Introduction

As sustainable green biocatalysts, enzymes exhibit high catalytic efficiency, strong substrate specificity, and environmental friendliness; they have been widely used in food processing, chemical industries, biopharmaceuticals, and environmental catalysis [1,2]. However, free enzymes frequently have disadvantages, such as poor recyclable quality, severe deactivation when used in industrial settings because of prolonged stirring, high temperatures, and inappropriate solvents, among other issues [3,4]. In recent years, there has been a growing interest in enzyme immobilization technology to overcome the challenges faced in the industrial application of enzymes [5,6].

Metal–organic framework materials (MOFs) have emerged as multifunctional porous crystal materials with high surface area, porosity, abundant coordination sites, tunable pore size, and functionality, which made them ideal candidates for enzyme immobilization carriers [7,8,9]. MOFs can create a stable microenvironment for enzyme molecules, which limits the leakage of enzyme molecules to a certain extent, and significantly improves the enzyme loading or the efficiency of substrate transfer [10]. Moreover, MOFs have proven to be able to enhance enzyme stability by maintaining the conformational structure of the enzyme and providing additional defense against unfavorable circumstances [11]. UiO-66-NH_2_ is a MOF material based on zirconium and 2-aminoterephthalic acid as the metal center and organic ligand, respectively, which displays excellent stability in strong acidic or alkaline solutions. Relevant studies have shown that the high specific surface area and porosity of amino-functionalized UiO-66 can enhance the loading capacity and mass transfer efficiency of the enzymes, simultaneously improving its stability and activity [12]. However, the robust interactions between enzymes and MOFs often restrict conformational freedom and typically diminish apparent activity in most cases [13]. In this case, the development of modifiers to regulate the interaction between enzymes and MOFs is highly desirable.

In recent years, ILs have been widely used as a novel carrier modifier in the field of enzyme immobilization due to their unique advantages [14]. In our previous studies, ILs have been demonstrated to be a series of excellent modifiers of chitosan, carbon nanotubes, and magnetic materials in enzyme immobilization [15,16]. Most recently, our team investigated IL-modified MOF (IL-NH_2_-MIL-101) for laccase immobilization, which confirmed that immobilized enzymes exhibited improved stability and activity, and achieved efficient pollutant degradation rates [17]. Moreover, Suo et al. [18] developed amino-functionalized IL-grafted magnetic MOF (ZIF-90) for the immobilization of porcine pancreatic lipase (PPL-ILs/MZIF-90), which demonstrated the high esterification efficiency of synthetic isoamyl acetate. The introduction of IL enhances the binding between the enzyme and the carrier, improves the flexibility of the carrier on the surface of the enzyme, and further alters the structural conformation of the lipase. Importantly, the diffusion restriction between the enzyme and the substrate was reduced, improving the microenvironment of the immobilized enzyme. In conclusion, different MOF materials modified with IL can be used for the immobilization of different enzymes. However, some reports on IL-modified MOF in *Candida rugosa* lipase (CRL) immobilization are limited.

**Scheme 1:**
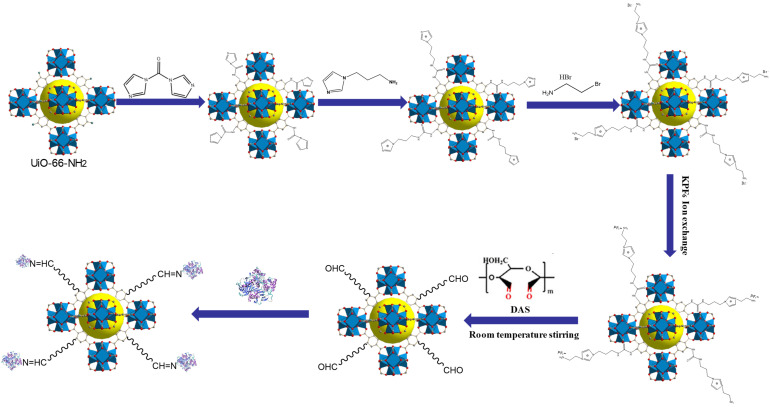
Schematic illustration of the synthesis of UiO-66-NH_2_-NIL-DAS and CRL immobilization.

CRL is nowadays one of the most widely used commercial lipases, which has drawn much attention for widespread application in hydrolysis, alcoholysis, aminolysis, and trans-esterification. However, free CRL is environmentally sensitive with low stability. Consequently, in the present study, a novel strategy for the modification of MOF was proposed for CRL immobilization. Furthermore, rich in active aldehyde groups, dialdehyde starch (DAS) has great biocompatibility and biodegradability, and is useful as a crosslinking agent for enzyme immobilization [19]. Hence, the IL-modified UiO-66-NH_2_ was synthesized, and a biocompatible biomacromolecule (DAS) was employed as a cross-linker between the lipase and the carrier-enhanced lipase activity and stability. This method was novel in that it integrated the advantages of the large specific surface and ease of modification of UiO-66-NH_2_ with the excellent stability of IL [20]. The introduction of IL into UiO-66-NH_2_ improved the carrier microenvironment and maintained the conformation of the enzyme, thereby increasing the stability and activity of the enzyme. The synthesis process of the composite and the immobilization procedure is illustrated in Scheme 1. This novel strategy provides a theoretical reference for the preparation of a novel and efficient MOF composite for immobilized lipase.

## 2. Results and Discussion

### 2.1. Characterization Analysis

**Figure 1:**
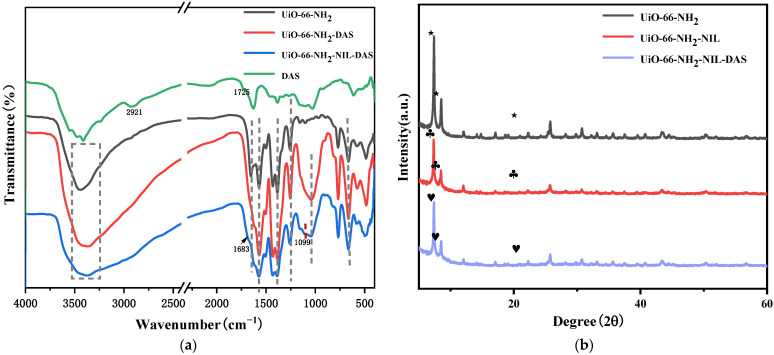
FT-IR (**a**) and XRD (**b**) spectra of the support carriers. The three different symbols represent the same peak positions for the three different carriers.

The FT-IR spectra of DAS, UiO-66-NH_2_, UiO-66-NH_2_-DAS, and UiO-66-NH_2_-NIL-DAS are displayed in Figure 1a. The characteristic peaks of DAS were observed at 2921 and 1725 cm^−1^, attributed to the stretching vibration of C–H and C=O of the aldehyde group, respectively [21]. The absorption bands at 3448 cm^−1^ and 1575 cm^−1^ were attributed to the symmetric stretching vibrations and bending vibrations of the -NH_2_ groups and N-H for three prepared carriers, respectively [22]. In Figure S2, the peak observed at 1384 cm^−1^ was attributed to the symmetric stretching vibration of O-C=O, which confirmed the presence of 2-aminoterephthalic acid in the framework [23]. Furthermore, the absorption peak at 1255 cm^−1^ was attributed to the stretching vibration of C-N on UiO-66-NH_2_ [24].The characteristic peaks at 658 cm^−1^ and 771 cm^−1^ contributed to the stretching vibrations of Zr-O and N-H, respectively [25,26]. The new peak was observed for UiO-66-NH_2_-NIL-DAS at 1099 cm^−1^ after the modification of UiO-66-NH_2_ with IL, which was attributed to the C-N stretching vibration within the imidazole moiety plane after the modification of IL [27]. Meanwhile, the new peak at 1045 cm^−1^ of UiO-66-NH_2_-NIL-DAS was attributed to the C-N in amide bonds, which indicated that NIL was successfully bound to UiO-66-NH_2_ [17]. In addition, the peak at 1683 cm^−1^ was attributed to the aldehyde group on the UiO-66-NH_2_-NIL-DAS, and the absorption peak at 1621 cm^−1^ was identified as the C=N vibration of the Schiff base formed by the reaction of aldehyde groups and amino groups [28]. It confirmed that DAS had been successfully bonded to UiO-66-NH_2_-NIL.

The XRD results of the carriers are shown in Figure 1b. The XRD pattern of UiO-66-NH_2_ exhibited characteristic peaks at 2θ = 7.4°, 8.5°, and 25.7°, corresponding to the (111), (200), and (600) planes of UiO-66-NH_2_. This suggested the successful preparation of UiO-66-NH_2_, which is consistent with the results of FT-IR [29,30]. After further modification by IL and DAS cross-linking, the carrier still maintained the same diffraction peaks, which indicated that the crystal structure of the carrier was well preserved. The peaks of UiO-66-NH_2_-NIL-DAS at 2θ = 7.4°, 8.5° and 25.7° maintained the characteristic peaks of UiO-66-NH_2_, demonstrating that the crystal structure of UiO-66-NH_2_ was not destroyed during the IL modification.

**Figure 2:**
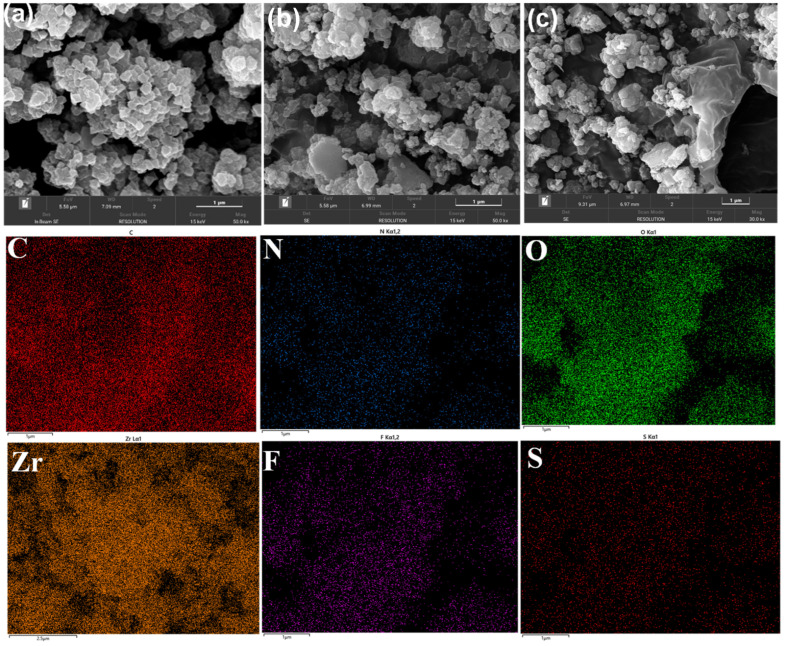
SEM and mapping images of UiO-66-NH_2_ (**a**), UiO-66-NH_2_-NIL-DAS (**b**), UiO-66-NH_2_-NIL-DAS@CRL (**c**).

The microstructure and morphology of the prepared three supports were characterized using SEM. As shown in Figure 2a, the UiO-66-NH_2_ exhibited regular octahedral crystals with a smooth surface, measuring approximately 200 nm in diameter [31]. Figure 2b shows the outcomes that were attained following the modifications of IL and DAS. The roughness of the UiO-66-NH_2_-NIL-DAS was observed to increase compared to the UiO-66-NH_2_, which is likely attributed to the modification with IL and DAS, providing evidence for the successful modification of UiO-66-NH_2_ by IL and DAS. Additionally, the surface of the support exhibited a white flocculent layer, primarily attributed to the interaction between UiO-66-NH_2_ and IL. Furthermore, SEM images (Figure 2c), revealed that the surface of the carrier was extremely rough, which can be attributed to the enzyme covalently agglomerating on the carrier. As shown in Figure S2a, there is no obvious change in the morphology and structure of the UiO-66-NH_2_ modified with DAS, and the results are consistent with the XRD results, indicating that the structure of the carrier material remains intact after DAS modification. Finally, the presence and distribution of the elements C, O, N, Zr, and F in UiO-66-NH_2_-NIL-DAS were confirmed by spectral analysis. It is worth noting that the F element is derived from IL, demonstrating the successful incorporation of both ILs and UiO-66-NH_2_.

**Figure 3:**
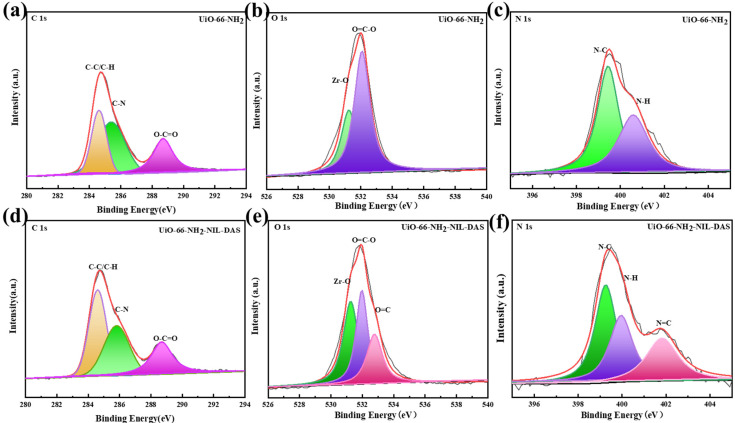
The XPS spectra of (**a**) C 1 s, (**b**) O 1 s, and (**c**) N 1 s core levels of the UiO-66-NH_2_-DAS; XPS spectra of (**d**) C 1 s, (**e**) O 1 s and (**f**) N 1 s core levels of the UiO-66-NH_2_-NIL-DAS.

The chemical states and chemical element composition of the carriers were analyzed by XPS. The full spectrum results are present in (Figure S3) and peak-splitting results of C, O, and N are shown in Figure 3. The results showed that the UiO-66-NH_2_-NIL-DAS carrier contains the C, N, O, Zr, and F elements, which was consistent with the results in the mapping images and confirmed the successful preparation of UiO-66-NH_2_-NIL-DAS. As shown in Figure 3a, the peaks in C 1 s were observed at 284.6 eV, 285.4 eV, and 288.7 eV corresponding to the C-C, O-C=O groups in 2-amino terephthalic acid, and the C-N group in amine [32,33]. The C-N bond binding energy on UiO-66-NH_2_-NIL-DAS was increased due to the changed chemical environment around the C atom of UiO-66-NH_2_ caused by the introduction of IL in Figure 3a. The high-resolution O 1 s peaks at 531.2 eV and 532.1 eV for UiO-66-NH_2_ and UiO-66-NH_2_-NIL-DAS in Figure 3b,e are assigned to Zr-O and O=C-O, respectively [34]. The successful cross-linking of DAS was also demonstrated by the observation of a C=O bond generated by the Schiff base reaction of DAS with the amino group on IL, with a binding energy at 532.8 eV [33]. The N 1 s spectrum of UiO-66-NH_2_ includes two distinct components, the N-C bond and the N-H bond. After IL modification, a new peak was observed at 401~402 eV, which was attributed to the N=C of the imidazole ring within IL, indicating the successful modification of IL [17]. Finally, the peak of F 1 s (Figure S3) was also observed for UiO-66-NH_2_-NIL-DAS, resulting from the exchange of the PF_6_^−^.

The thermal stability of the carriers was evaluated by TGA, and the results are shown in Figure S4. According to the curve of UiO-66-NH_2_, there are three main stages of weightlessness, which is consistent with some previous literature [35,36]. The first stage of weight loss occurred before 180 °C, which was attributed to the evaporation of adsorbed water and DMF on the surface of UiO-66-NH_2_. The second stage was observed at 180–370 °C, which was relative to the decomposition of Zr_6_O_4_(OH)_4_ to form Zr_6_O_6_. Finally, the framework of Zr_6_O_6_ underwent fracture and dehydration prior to oxidating to ZrO_2_ in the third stage when the temperature exceeded 370 °C. There was almost no weight loss in UiO-66-NH_2_ within 100~200 °C phase in Figure S4a, while the weight loss in UiO-66-NH_2_-NIL was almost the same as that of UiO-66-NH_2_-NIL-DAS, which was about 4.2%. They were significantly higher than UiO-66-NH_2_, suggesting that was caused by the thermal decomposition of IL at 100~200 °C. From the TGA graph (Figure S5), the residual mass of UiO-66-NH_2_ is 62.5%, whereas the residual mass of UiO-66-NH_2_ is 60.5%. The weight loss of UiO-66-NH_2_-NIL was higher than that of UiO-66-NH_2_, which indicated the successful grafting of IL. The weight loss of UiO-66-NH_2_-NIL-DAS was slightly higher than that of UiO-66-NH_2_-NIL, which can be attributed to the decomposition of DAS on the surface of UiO-66-NH_2_-NIL-DAS [28]. The results indicated that UiO-66-NH_2_-NIL-DAS had been successfully prepared with good thermal stability.

The porous features of UiO-66-NH_2_ and UiO-66-NH_2_-NIL-DAS were investigated by nitrogen adsorption isotherms, shown in Figure S4b. Notably, two groups showed I-type isothermal curves, indicating that the samples are mainly composed of micropores, which is consistent with the results reported in the literature [37]. The specific surface areas of UiO-66-NH_2_ and UiO-66-NH_2_-NIL-DAS were determined to be 658.63 and 348.47 m^2^/g, and the pore volume were determined to be 0.26 and 0.16 cm^3^·g^−1^, respectively. The reduction in the specific surface area was probably due to the occupation of pore structure by the introduced IL, which confirmed the successful synthesis of the carriers. Nevertheless, the maintained high specific surface area and open pores of UiO-66-NH_2_-NIL-DAS provided favorable conditions for enzyme immobilization.

**Figure 4:**
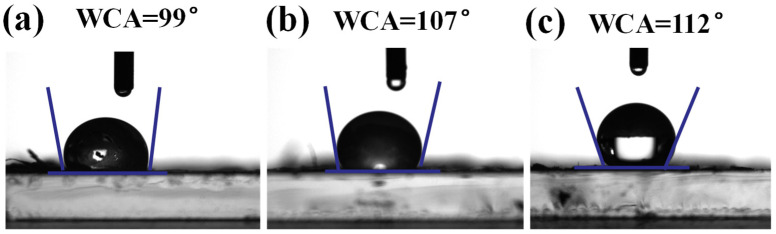
The water contact angle of (**a**) UiO-66-NH_2_, (**b**) UiO-66-NH_2_-DAS and (**c**) UiO-66-NH_2_-NIL-DAS.

The presence of a lid can diminish the catalytic activity of lipase by preventing the substrate from binding to the active center. When lipase is positioned at the hydrophobic interface, its adhesion through hydrophobic interaction triggers the opening of the lid on the active center, thereby facilitating substrate entry into the enzyme active site and consequently boosting lipase activity [38,39]. To study the hydrophobicity of the carriers, the water contact angles of three carrier materials (UiO-66-NH_2,_ UiO-66-NH_2_-DAS, and UiO-66-NH_2_-NIL-DAS) were investigated (Figure 4). The water contact angle of UiO-66-NH_2_-DAS and UiO-66-NH_2_-NIL-DAS is significantly higher than that of UIO-66-NH_2_, showing that the interaction between IL and UiO-66-NH_2_ increases the hydrophobicity of UiO-66-NH_2_-NIL-DAS. Meanwhile, the increased hydrophobicity of UiO-66-NH_2_-NIL-DAS makes lipase attach to the hydrophobic pocket more easily, and this interfacial activation facilitates the opening of the lid region of the lipase active center and promotes substrate access to the active center [38].

### 2.2. The Optimization Conditions of CRL Immobilized on UiO-66-NH_2_-DAS and UiO-66-NH_2_-NIL-DAS

**Figure 5:**
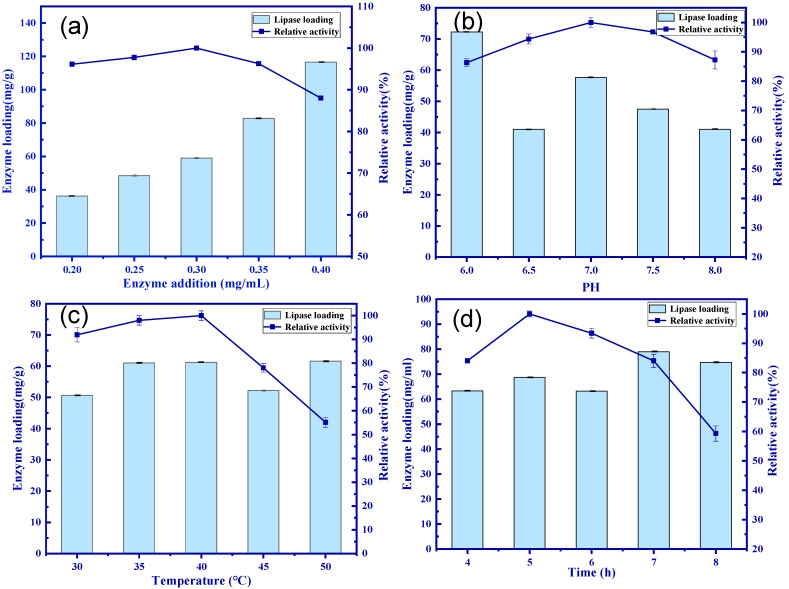
The effects of the initial lipase concentration (**a**), pH (**b**), temperature (**c**), and immobilization time (**d**).

The effects of immobilization conditions, including enzyme addition, pH, temperature, and time, were investigated, and results are shown in Figure 5 and Figure S1. As shown in Figure 5a, the relative activity gradually increased to a peak at the initial addition of 0.3 mg/mL of lipase concentration. After that, the relative activity of the immobilized enzyme started to decrease even if the amount of enzyme was slightly increased further, which can be attributed to the fact that the overloading of lipase may lead to a change in the stable conformation of lipase, which results in the blocking or destroying of its active site. Meanwhile, the optimal pH of the immobilized lipase is 7.0, which was the same as the optimal reaction pH of the free CRL (Figure 5b). The optimal time and immobilization temperature was 5 h and 40 °C (Figure 5c,d), respectively. Moreover, as shown in Figure S1, the optimal immobilization condition of lipase on UiO-66-NH_2_-DAS was 0.25 mg/mL lipase, 40 °C, pH = 7.0, and 5 h. In summary, the optimization conditions are outlined in Table S1.

The immobilized CRL results of UiO-66-NH_2_-DAS and UiO-66-NH_2_-NIL-DAS are summarized in Table 1. As shown in Table 1, the CRL loading capacity of UiO-66-NIL-DAS was 65 mg/g, which was higher than that reported in the literature for the covalent immobilization of CRL on a DAC-modified organic-inorganic nanocomposite carrier [4]. Moreover, the specific activity and activity recovery of UiO-66-NH_2_-NIL-DAS immobilized lipase were higher than that of UiO-66-NH_2_-DAS. The enhanced catalytic activity of UiO-66-NH_2_-NIL-DAS is mainly attributed to the introduction of IL to improve the immobilization microenvironment, which is conducive to maintaining the conformational integrity and active state of the enzyme [40,41]. Therefore, UiO-66-NH_2_-NIL-DAS is the most promising carrier for enzyme immobilization.

### 2.3. Effects of ILs-Modified Carrier on the Optimal pH and Temperature of Lipase

**Figure 6:**
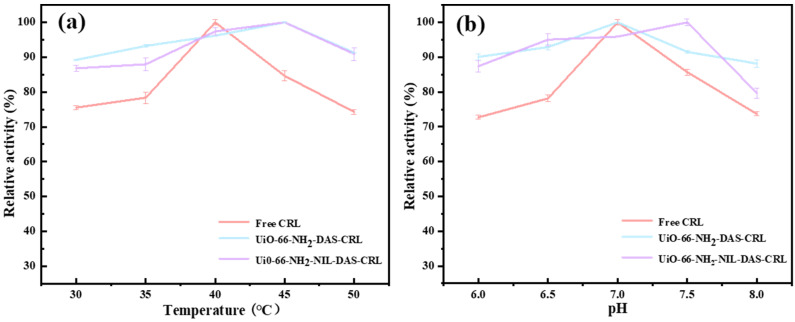
Effects of temperature (**a**) and pH (**b**) on free CRL, UiO-66-NH_2_-DAS@CRL, and UiO-66-NH_2_-NIL-DAS@CRL.

To investigate the immobilization performance of a ILs-modified carrier, the change in the optimal pH and temperature of CRL and immobilized CRL was investigated. As shown in Figure 6a, we screened the optimal reaction temperature for CRL, UiO-66-NH_2_-DAS@CRL, and UiO-66-NH_2_-NIL-DAS@CRL at pH = 7. The optimal reaction temperature of UiO-66-NH_2_-NIL-DAS@CRL and UiO-66-NH_2_-DAS@CRL was 45 °C, which is higher than that of free CRL. The better temperature tolerance of immobilized CRL is indicated by the variation in optimum temperature. After immobilization, the optimum temperature of the enzyme was further increased, but with the temperature increasing, the structure of the enzyme was disrupted, leading to enzyme inactivation. Subsequently, the screening of optimal reaction pH for CRL, UiO-66-NH_2_-DAS@CRL, and UiO-66-NH_2_-NIL-DAS@CRL at their optimal temperature is shown in Figure 6b. The UiO-66-NH_2_-NIL-DAS@CRL has a higher optimum pH compared to the free CRL, demonstrating that the sensitivity of immobilized CRL to pH decreased slightly. This may be attributed to the reduced effect of lower H^+^ concentration on the conformation of the immobilized lipase, which made the immobilized lipase have higher activity at high pH values [42].

### 2.4. Stability of Free CRL and Immobilized CRL

**Figure 7:**
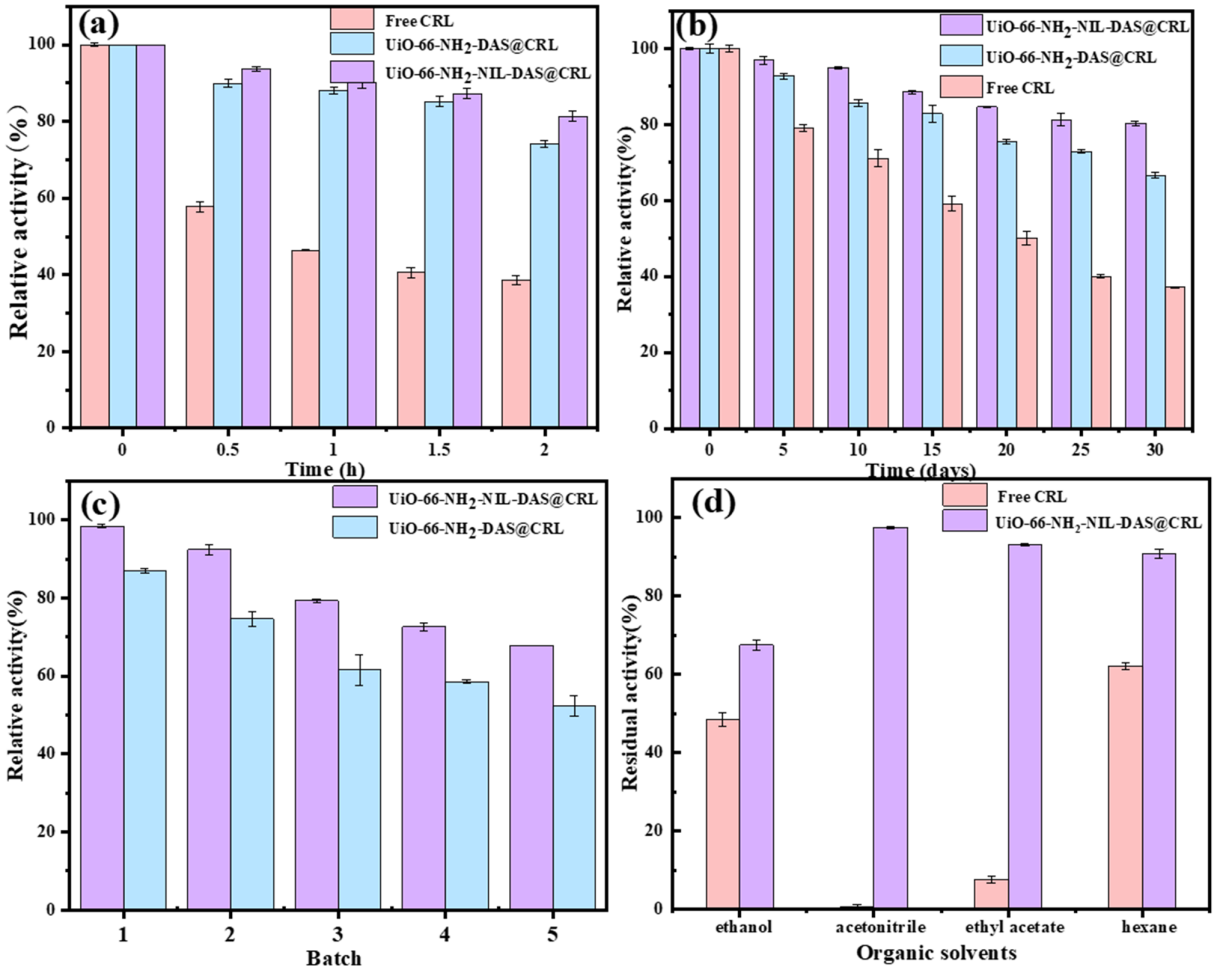
Thermal stability (**a**), and storage stability (**b**) of free CRL, UiO-66-NH_2_-DAS@CRL and UiO-66-NH_2_-NIL-DAS@CRL, (**c**) Reuse of the UiO-66-NH_2_-DAS@CRL and UiO-66-NH_2_-NIL-DAS@CRL, (**d**) Organic solvent tolerance of free CRL and UiO-66-NH_2_-NIL-DAS@CRL.

The thermal stability of immobilized enzymes is a critical issue to be considered to maintain the stability of enzymatic reactions [43]. As shown in Figure 7a, the activities of free CRL, UiO-66-NH_2_-DAS@CRL and UiO-66-NH_2_-NIL-DAS@CRL have declined to varying degrees with increasing incubation time. The activity-reduced rate of immobilized enzymes was significantly decreased compared with free CRL. The catalytic activity of free CRL decreased significantly from 0 min to 30 min, and only 38.6% of residual activity remained at 120 min. The residual activity of immobilized enzymes (UiO-66-NH_2_-DAS@CRL and UiO-66-NH_2_-NIL-DAS@CRL) remained at 89.6% and 93.8% after 30 min, respectively. The results confirmed that the activity of UiO-66-NH_2_-NIL-DAS@CRL was higher at any moment at 60 °C, indicating that the modification of ILs and macromolecular DAS enhanced the thermal resistance of the immobilized enzymes. Compared to the CRL immobilized on the poly(VAc-DVB) microsphere (p-MS) studied by Li et al., UiO-66-NH_2_-NIL-DAS@CRL has better thermal stability [44].

The three enzymes were placed in a refrigerator at 4 °C to store for 30 days, and residual enzyme activity was measured every 5 days. As shown in Figure 7b, the activity of the immobilized lipase decreased more slowly than that of free lipase. After 30 days of storage, the free CRL retained only 37.2% of its initial activity, whereas the UiO-66-NH_2_-NIL-DAS@CRL retained approximately 80.5% of its initial activity. When Jiang et al. studied CRL immobilized on microcapsules stored at 4 °C, the immobilized CRL retained 81% of its activity, and the activity of free CRL decreased rapidly [45]. Compared to the immobilized CRL studied by Jiang et al., the UiO-66-NH_2_-NIL-DAS@CRL we studied retained more stability in long-term storage.

Reusability is an important index of the continuous utilization of immobilized enzymes. The reusability of UiO-66-NH_2_-DAS@CRL and UiO-66-NH_2_-NIL-DAS@CRL was investigated, and shown in Figure 7c. The activity of the immobilized enzyme was 52.0% and 68.0% after five applications, respectively. Therefore, UiO-66-NH_2_-NIL-DAS@CRL has a better reusability. This suggested that the introduction of ILs enhanced the interaction force with the support, which was conducive to providing a good microenvironment for the enzymes and reducing the inactivation rate of the enzymes. It was found that the activity of covalently immobilized CRL on UiO-66-NH_2_-NIL-DAS was significantly higher than the 42.15% retention of immobilized CRL previously reported by Jafarian et al. after five cycles [46].

Lipases can be used for biocatalytic reactions, not only in the aqueous phase, but can also be applied to biocatalysis in non-aqueous media. The effect of organic solvents on the stability of free and immobilized CRL was investigated, taking into account practical application values. After treatment in an organic solvent for 2 h, the residual activity of free CRL and UiO-66-NH_2_-NIL-DAS@CRL was determined, and the results were shown in Figure 7d. The resistance of UiO-66-NH_2_-NIL-DAS@CRL to ethanol, acetonitrile, ethyl acetate, and hexane was significantly more stable than that of free CRL. In particular, the residual activity of UiO-66-NH_2_-NIL-DAS@CRL was the highest in acetonitrile, while free CRL was completely inactive. This was attributed to the covalent bond between the CRL and the carrier that preserved the integrity of the lipase. The introduction of IL maintained the rigidity of the lipase and improved the organic solvent tolerance of the lipase. Cea et al. evaluated the tolerance of CRL immobilized on biochar in organic solvents (acetonitrile, hexane, etc.) and found that only 35.0% and 65.0% of the activity was retained in acetonitrile and hexane, respectively [47]. Thus, the UiO-66-NH_2_-NIL-DAS@CRL studied has a better tolerance to organic solvents.

### 2.5. Michaelis–Menten Kinetic Parameters of Free CRL and Immobilized CRL

**Figure 8:**
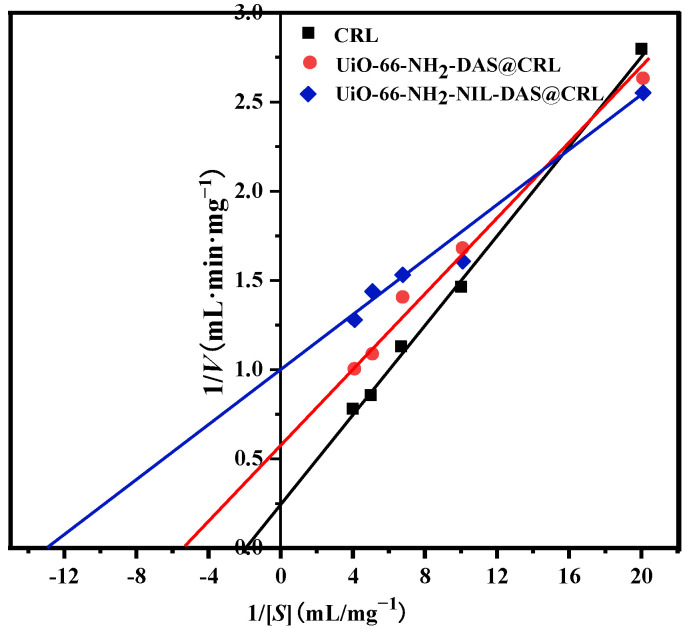
Lineweaver–Burk plots for CRL, UiO-66-NH_2_-DAS@CRL, and UiO-66-NH_2_-NIL-DAS@CRL.

The kinetic parameter is a crucial evaluation index of the immobilized enzymes. The *K_m_* and *V_max_* were investigated by plotting the Lineweaver–Burk plot of immobilized lipase (Figure 8), and the results are shown in Table 2. The results revealed that the affinity of immobilized CRL (UiO-66-NH_2_-DAS@CRL and UiO-66-NH_2_-NIL-DAS@CRL) toward the substrate was increased compared with free CRL, indicating a higher affinity of the immobilized enzyme toward the substrate. The kinetic parameters of UiO-66-NH_2_-DAS@CRL and UiO-66-NH_2_-NIL-DAS@CRL showed that the introduction of IL decreased the Km value of UiO-66-NH_2_-NIL-DAS@CRL compared with UiO-66-NH_2_-DAS@CRL. The decrease in the *K_m_* value may be attributed to the fact that the surface of the nanomaterials has more reactive sites, making the substrate more accessible to the active site, suffering minimal mass transfer limitations, and increasing the nucleophilicity with the substrate. These facilitated the substrate to enter the reaction center. Moreover, the decreased *V_max_* value of UiO-66-NH_2_-NIL-DAS@CRL could be attributed to the introduction of DAS macromolecules, which increased its spatial site resistance and reduced the chance of enzyme contact with the substrate. The increase in the value of *V_max_/K_m_* was favorable to the increase of hydrolytic activity. Therefore, the UiO-66-NH_2_-NIL-DAS carrier was more beneficial to the improvement of lipase activity.

## 3. Material and Method

### 3.1. Materials

*Candida rugosa* lipase (type VII) was purchased from Sigma-Aldrich (St Louis, MO, USA). Zirconium chloride (ZrCl_4_) and *N-*(3-Aminopropyl)-imidazole were purchased from Shanghai Aladdin Biochemical Technology Co., Ltd. (Shanghai, China). *N*,*N*′-Carbonyldiimidazole (CDI, 99.5%), *N*,*N*-dimethylformamide (DMF, 99.5%), 1,8-diazabicyclo[5.4.0]undec-7-ene (DBU, 98.0%), 2-aminoterephthalic acid and dimethyl sulfoxide (DMSO) were purchased from Aladdin Industrial Corporation. Potassium hexafluorophosphate (KPF_6_) was supplied by Energy Chemical (Shanghai, China). 2-Bromoethylamine hydrobromide was provided by 9 Ding Chemistry (Shanghai, China) and dialdehyde starch (DAS) was purchased from Tai’an Jinshan Modified Starch Co., Ltd. (Tai’an, China).

### 3.2. Characterization

Fourier transform infrared (FT-IR, Nicolet iS5) spectroscopy analysis was carried out to determine the structure of surface functional groups in the wavelength range of 400–4000 cm^−1^. The thermalgravimetric analysis was investigated using a TGA 550 instrument under an N_2_ atmosphere, from room temperature to 800 °C. The crystal structure of the carriers were characterized by utilizing a D8 Advance X-ray diffractometer (XRD). Scanning electron microscope (SEM) was performed on a FE-SEM SU8200 instrument (Japan). The hydrophobicity of carriers was tested using a contact angle meter (JY-82C). Surface elements and chemical states were analyzed using an X-ray photoelectron spectrometer (XPS).

### 3.3. Synthesis of UiO-66-NH_2_ and ILs-Modified UiO-66-NH_2_

UiO-66-NH_2_ was synthesized by a solvothermal method. Firstly, ZrCl_4_ (0.48 g) and NH_2_-BDC (0.36 g) were dispersed in 60 mL of *N*,*N*-dimethylformamide (DMF) and dissolved through sonication for 15 min, and the mixture was stirred for 30 min. The resulting solution was then transferred to a Teflon container and heated at 120 °C for 24 h. Subsequently, after cooling the suspension to room temperature, the mixture was centrifuged (10,000 rpm, 10 min). The mixture was washed several times with DMF, and finally with ethanol, and dried at 120 °C for 12 h.

The ILs-modified UiO-66-NH_2_ (UiO-66-NH_2_-NIL) was synthesized following a previously reported method with some modifications [48]. Typically, UiO-66-NH_2_ (0.8 g) and DBU (3 mg) were dispersed in toluene (20 mL) and stirred in an ice bath for 1 h, and then CDI (286 mg) was added and stirred at room temperature for 12 h. Then, the intermediate product was obtained by filtration, ethanol washing, and drying in a vacuum oven at 80 °C. Subsequently, the obtained solid (UiO-66-NH_2_-CDI) was dispersed in DMSO (50 mL), and 0.45 g of 1-(3-aminopropyl)-imidazole was slowly added dropwise to the reaction system, which was further stirred at room temperature for 12 h. The acquired solid (UiO-66-NH_2_-IM) was filtrated and washed with ethanol. Then, the solid was transferred to acetonitrile (30 mL) to disperse and dissolve. Subsequently, 6.5 mmol of 2-Bromoethylamine hydrobromide was dissolved in 20 mL of acetonitrile and neutralized with NaOH for 30 min, then the mixture was poured into the above solution. The reaction was conducted under nitrogen protection at 80 °C for 24 h. The slurry was washed with water and ethanol, followed by dispersion in water containing KPF_6_ (1 g) for 12 h of ion exchange. The obtained UiO-66-NH_2_-NIL was washed three times with water and ethanol and dried in an oven under a vacuum.

### 3.4. Synthesis of UiO-66-NH_2_-NIL-DAS

The UiO-66-NH_2_-NIL (30 mg) was dispersed in 4 mL of different concentrations of DAS solution prepared from phosphate buffer solution and stirred at room temperature for 12 h. The mixture was washed with purified water and dried for use. The final product was designated as UiO-66-NH_2_-NIL-DAS. The optimum concentration of DAS after optimization was 1.5%.

### 3.5. Lipase Immobilization

A quantity of lipase was dissolved in phosphate buffer solution and centrifuged to obtain a homogeneous enzyme solution; the obtained supernatant was utilized for subsequent experiments.

UiO-66-NH_2_-NIL-DAS (50 mg) or UiO-66-NH_2_-DAS (50 mg) was dispersed in a buffer solution (pH = 7) containing different lipase concentrations (0.20–0.40 mg/mL) and incubated at different temperatures (30–50 °C) for 4–8 h. The effects of enzyme concentration, pH, temperature, and the immobilization time of the carriers (UiO-66-NH_2_-NIL-DAS (Figure 5) and UiO-66-NH_2_-DAS (Supplementary Materials)) on relative activity and loading capacity were investigated. After immobilization, the immobilized lipase was centrifuged and washed several times with the corresponding buffer solution. Finally, the resulting immobilized enzyme was freeze-dried and stored at 4 °C. The protein concentration was determined according to the Bradford method [49].

After the optimization of the immobilized conditions, the two novel carriers were immobilized under their respective optimal conditions. Experimental results were determined by calculating the average of three data values for each measurement. All measurements were repeated three times, the loading capacity and immobilization efficiency were calculated with the following formulas:

(1) $$\mathrm{CRL}\;\mathrm{loading}\;\left(mg/g\right)=\frac{C_{i}\times V_{i}-C_{f}\times V_{f}}{M_{f}}$$

(2) $$\mathrm{Immobilization}\;\mathrm{efficiency}\;\left(\%\right)=\frac{C_{i}\times V_{i}-C_{f}\times V_{f}}{C_{i}\times V_{i}}\times 100$$

where *C_i_* (mg/g) is the initial protein concentration of CRL, the *C_f_* (mg/mL) is the final protein concentration in the supernatant and washing solution after immobilization, the *M_f_* (g) is the quality of the support, the *V_i_* is the total volume of enzyme solution added to the system and the *V_f_* (mL) is the volume of the supernatant and washing liquid.

### 3.6. Lipase Activity Assay

Lipase activity was assessed by measuring the ability to hydrolyze p-nitrophenyl palmitate (*p*-NPP) [50]. Enzyme activity (1 U) is defined as the amount of enzyme needed to catalyze the hydrolysis of *p*-NPP to produce 1 μmol of p-nitrophenol per minute under a certain temperature and pH value. All measurements were repeated at least thrice, and the activity of the immobilized CRL was calculated with the following formula:

(3) $$\mathrm{Expressed}\;\mathrm{activity}\;\left(U/g\right)=\frac{A\times 10^{6}\times V_{t}}{\varepsilon \times t\times m_{1}}$$

(4) $$\mathrm{Specific}\;\mathrm{activity}\;\left(U/g\right)=\frac{A\times 10^{6}\times V_{t}}{\varepsilon \times t\times m_{2}}$$

where *A* is the absorbance of the samples, *V_t_* (L) is the volume of the reaction system, *ε* (L/mol^−1^·cm^−1^) is the molar extinction coefficient of *p*-nitrophenol, *t* (min) is the reaction time, *m*_1_ (g) is the mass of immobilized CRL and *m*_2_ (g) is the protein contained in the immobilized CRL. Relative activity and residual activity were calculated using:

(5) $$\mathrm{Relative}\;\mathrm{activity}\;\left(\%\right)=\frac{A_{t}}{A_{max}}\times 100$$

(6) $$\mathrm{Residual}\;\mathrm{activity}\;\left(\%\right)=\frac{A_{t}}{A_{i}}\times 100$$

where *A_t_* is the absorbance of the samples, *A_max_* is the maximum absorbance, and *A_i_* is the initial absorbance of the samples.

Activity recovery (%): Ratio of immobilized lipase activity to free enzyme activity used for immobilization.

(7) $$\mathrm{Activity}\;\mathrm{recovery}\;\left(\%\right)=\frac{\mathrm{Total}\;\mathrm{expressed}\;\mathrm{activity}\;\mathrm{of}\;\mathrm{immobilized}\;\mathrm{enzyme}}{\mathrm{Total}\;\mathrm{expressed}\;\mathrm{activity}\;\mathrm{of}\;\mathrm{free}\;\mathrm{enzyme}}\times 100$$

### 3.7. Optimum Temperature and Optimum pH for Free and Immobilized CRL

The effect of pH on the activity of free and immobilized lipase was investigated by determining the activity in phosphate buffer solution (0.1 M) with pH (6.0–8.0) at 40 °C. The effect of temperature on the activity of free and immobilized lipase was investigated at their respective optimal pH conditions from 30 °C to 50 °C. The maximum activity of the highest free and immobilized CRL was defined as 100%.

### 3.8. Enzyme Kinetics Study of Free and Immobilized CRL

To measure the Michaelis–Menten constant (*K_m_*) and the maximum enzymatic reaction rate (*V_max_*) of immobilized lipase, a certain amount of immobilized CRL was added to 5 mL of *p*-NPP solution in the concentration range of 0.05–0.25 mg/mL, and the reaction was carried out at their respective optimum pH and temperature for 10 min. Finally, the *K_m_* and *V_max_* values are calculated using the Lineweaver–Burk plot:

(8) $$\frac{1}{V}=\frac{K_{mapp}}{V_{max}\left[S\right]}+\frac{1}{V_{max}}$$

where *V* and *V_max_* represent the initial rate and the maximum rate of the enzymatic reaction, respectively, and [*S*] is the substrate concentration.

### 3.9. Study on Stability of Free CRL and Immobilized CRL

To assess thermal stability, the free and immobilized CRL was incubated at 60 °C for 2 h and the enzyme activity was measured at the respective optimum condition every half hour. To further investigate the storage stability, free and immobilized CRL were refrigerated at 4 °C for 30 days. The residual enzyme activity was measured every 5 days at the respective optimal condition. The initial activity of the enzyme was defined as 100%.

To determine the reusability of immobilized CRL, the immobilized lipase was reused several times at their respective optimum temperature and pH. After each reaction cycle was completed, the immobilized enzyme was centrifuged, washed with phosphate buffer solution (pH = 7.0, 0.1 M), and reused for the next cycle. The activity was investigated at the respective optimum condition, and the initial activity was defined as 100%.

To determine the tolerance in organic solvents, CRL and immobilized CRL were soaked in ethanol, acetonitrile, ethyl acetate, and hexane for 2 h at room temperature. The obtained solid was isolated by centrifugation, filtration and repeatedly washed with buffer. Afterward, the residual catalytic activities were measured.

## 4. Conclusions

In this research, UiO-66-NH_2_ was successfully modified with amino-functionalized IL and applied to the immobilization of CRL. The covalent bond formed between IL-modified MOF and DAS strengthened the interaction between the carrier and lipase, which was conducive to the maintenance of the spatial conformation of the lipase and the enhancement of its catalytic activity. These results showed that the prepared immobilized CRL had good stability, catalytic activity, and reusability. The water contact angle test confirmed that UiO-66-NH_2_-NIL-DAS was more hydrophobic and more conducive to the opening of the lid of the lipase activity center. The kinetic parameters showed that UiO-66-NH_2_-NIL-DAS@CRL had a strong affinity for the substrate and high catalytic efficiency. In conclusion, IL-modified MOF offered a broad potential for enzyme immobilization. We expect that the strategy of enzyme immobilization using IL-modified MOF will provide more applications for the high production of efficient biocatalysts.

## Figures and Tables

**Table 1:** Results of the immobilized lipase.

Support	Lipase Loading (mg/g)	Specific Activity (U/g)	Activity Recovery (%)
UiO-66-NH_2_-DAS	62.4	1918	52.9
UiO-66-NH_2_-NIL-DAS	65.0	2872	79.3

**Table 2:** Results of the immobilization process of CRL.

Support	*K_m_* (mg/mL)	*V_max_* (mg/(mL·min))	*V_max_*/*K_m_*
CRL	0.51	4.02	7.88
UiO-66-NH_2_-DAS@CRL	0.181	1.702	9.4
UiO-66-NH_2_-NIL-DAS@CRL	0.077	1.01	13.12

## Data Availability

The original contributions presented in the study are included in the article/Supplementary Materials, further inquiries can be directed to the corresponding authors.

## Supplementary Materials

The following supporting information can be downloaded at: https://www.mdpi.com/article/10.3390/molecules29102381/s1, Figure S1: Effects of initially added lipase concentration (a), pH (b), temperature (c), and immobilization time (d) on lipase loading and relative activity of UiO-66-NH_2_-DAS carrier; Figure S2: SEM images of UiO-66-NH_2_-DAS(a); Figure S3: XPS survey scans of UiO-66-NH_2_ and UiO-66-NH_2_-NIL-DAS; Figure S4: TGA (a) and N_2_ adsorption–desorption curve (b) of the three prepared carries; Table S1: Optimal immobilization conditions for carriers.
