# Supplementary material for: Functionalized Ionic Liquids-Modified Metal–Organic Framework Material Boosted the Enzymatic Performance of Lipase
Source: Molecules. 2024 May 18;29(10):2381. doi: 10.3390/molecules29102381 (PMC11124374; doi:10.3390/molecules29102381)
Supplement: Supplementary file 1 [file molecules-29-02381-s001.zip › molecules-2989678-supplementary.pdf]

Supporting information

Optimal immobilization conditions

In the process of optimizing the immobilization conditions, the lipase activity was carried out at 40 ℃ and pH=7. The maximum lipase activity was defined as 100%.

1. Lipase concentration

   The carriers (UiO-66-NH_2-DAS or UiO-66-NH_2-NIL-DAS) were disperseded in the mixture (10 mL) containing phosphate buffer solution (0.1 M, pH=7) and different volumes of lipase solution (4-8 mL). Then the system was incubated at 30 ℃ for 4 h. The immobilized lipase was washed several times. Finally, the activity and loading capacity were examined.

2. pH

   The UiO-66-NH_2-DAS was sonicated in the mixture (10 mL) containing 5 mL phosphate buffer solution (0.1 M, pH=6-8) and 5 mL lipase concentration. The system was incubated at 30 ℃ for 4 h. The UiO-66-NH_2-NIL-DAS was sonicated in the mixture (10 mL) containing 4 mL phosphate buffer solution (0.1 M, pH=6-8) and 6 mL lipase concentration. Then the system was incubated at 30 ℃ for 4 h. The immobilized lipase was washed several times. Finally the activity and loading capacity were examined.

3. Temperature

   The supports (UiO-66-NH_2-DAS and UiO-66-NH_2-NIL-DAS) were sonicated in the mixture (10 mL) containing phosphate buffer solution (0.1 M, pH=7) and their respective lipase solution. Finally, the suspension was incubated at 30-50 ℃ for 4 h. The immobilized lipase was washed several times. Finally, the activity and loading capacity were examined.

4. Time

   The supports (UiO-66-NH_2-DAS and UiO-66-NH_2-NIL-DAS) were sonicated in the mixture (10 mL) containing phosphate buffer solution (0.1 M, pH=7) and their respective lipase solution. Finally, the suspension was incubated at 40 ℃ for 4-8 h. The immobilized lipase was washed several times. Finally, the activity and loading capacity were examined.

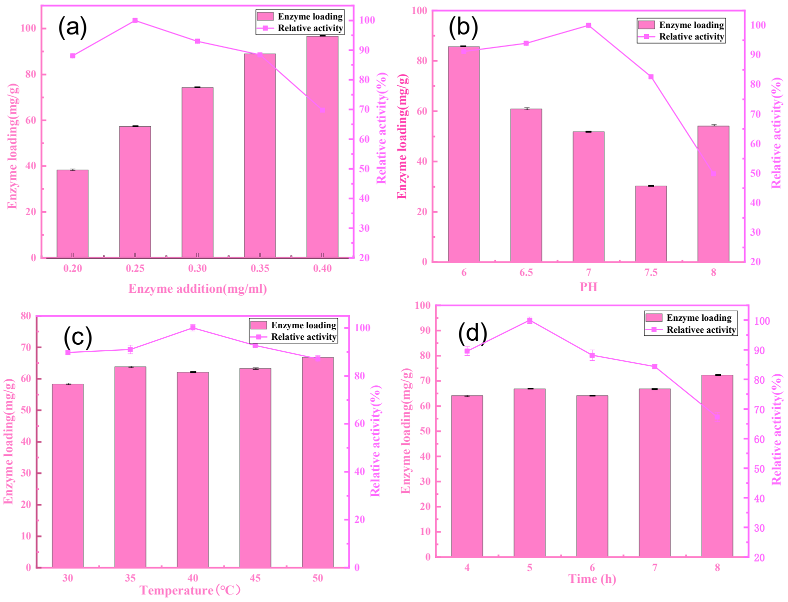

Figure S1. Effects of initially added lipase concentration (a), pH (b), temperature (c), and immobilization time (d) on lipase loading and relative activity of UiO-66-NH_2-DAS carrier.

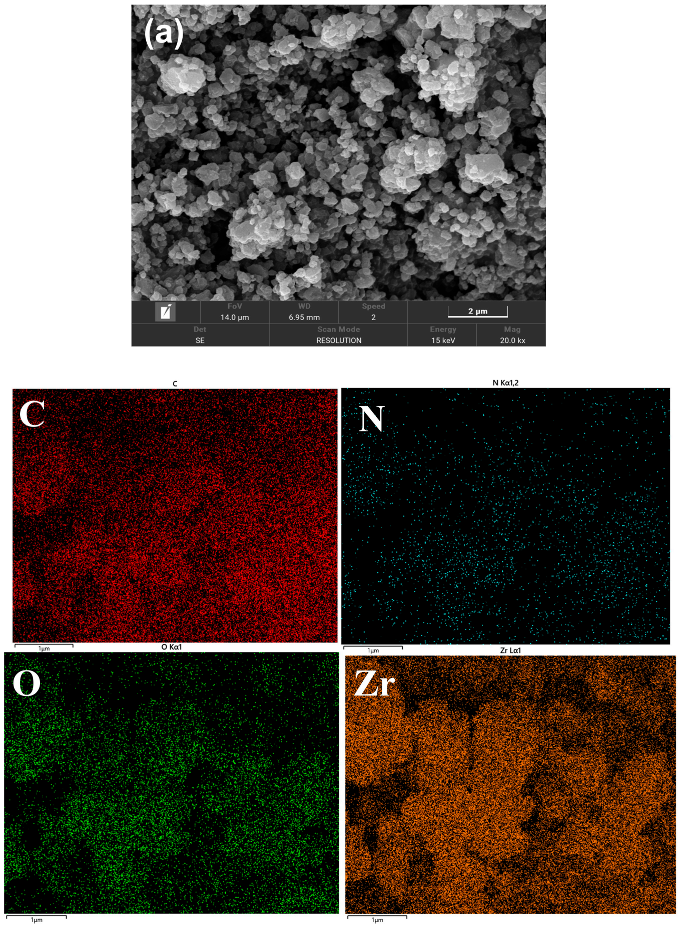

Figure.S2 SEM images of UiO-66-NH_2-DAS(a).

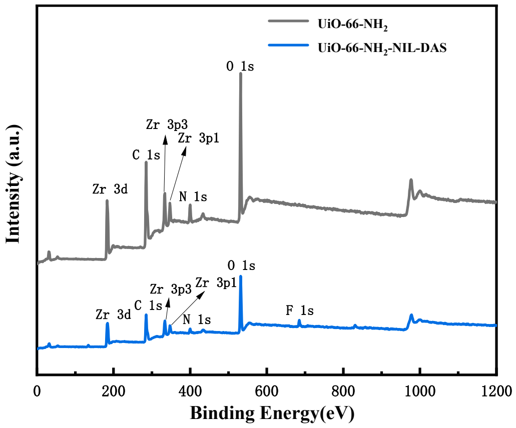

Figure.S3 XPS survey scans of $UiO-66-NH_2$ and $UiO-66-NH_2$-NIL-DAS.

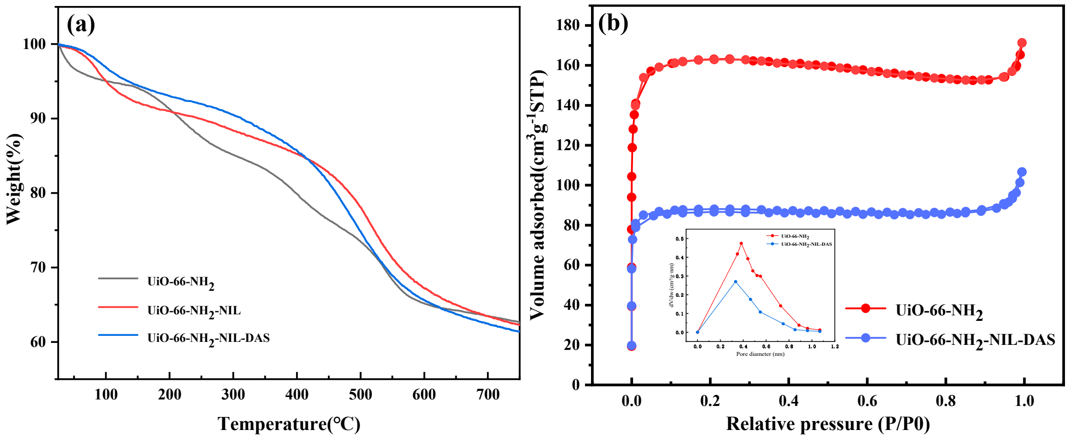

Figure S4. TGA (a) and N_2 adsorption-desorption curve (b) of the three prepared carries.

Table S1. Optimal immobilization conditions for carriers

Supports	Lipase concentration(mg/mL)	pH	Temperature (℃)	Immobilized time(h)
$UiO-66-NH_2$-DAS	0.25	7.0	40	5.0
$UiO-66-NH_2$-NIL-DAS	0.30	7.0	40	5.0
